# Supplementary material for: Multi-locus and long amplicon sequencing approach to study microbial diversity at species level using the MinION™ portable nanopore sequencer
Source: Gigascience. 2017 Jun 12;6(7):1–12. doi: 10.1093/gigascience/gix043 (PMC5534310; doi:10.1093/gigascience/gix043)
Supplement: GIGA-D-16-00108_Original_Submission.pdf [file gix043_GIGA-D-16-00108_Original_Submission.pdf]

Manuscript
Click here to download Manuscript Ultra-long_amplicon_sequencing_manuscript_Gigascience.docx
Click here to view linked References

Multi-locus and ultra-long amplicon sequencing approach to study microbial diversity at species level using the MinION^{TM} portable nanopore sequencer

Alfonso Benítez-Páez*, Yolanda Sanz

Microbial Ecology, Nutrition & Health Research Unit. Institute of Agrochemistry and Food Technology Institute (IATA-CSIC),Valencia, Spain.

*Corresponding author
C. Catedràtic Agustín Escardino Benlloch, 7.
46980 Paterna-Valencia
Spain
Tel: +34 963 900 022 ext. 2129.
Email: abenitez@iata.csic.es.

Abstract

Background: The miniaturised and portable DNA sequencer MinION™ has demonstrated great potential in different analyses such as genome-wide sequencing, pathogen outbreak detection and surveillance, human genome variability, and microbial diversity. In this study, we tested the ability of the MinION™ platform to perform ultra-long amplicon sequencing in order to design new approaches to study microbial diversity using a muli-locus approach.

Results: Using R9 chemistry, we generated more than 17,000 reads in a single sequencing run of 40h. After compiling a robust database by parsing and extracting the *rrn* bacterial region from more than 67,000 complete or draft bacterial genomes, we were able to demonstrate that the data obtained during sequencing of the ultra-long amplicon in the MinION™ device was sufficient to study two mock microbial communities in a multiplex manner and to almost completely reconstructthe microbial diversity contained in the HM-782D and D6305 mock communities.

Conclusions: Although nanopore-based sequencing produces reads with lower per-base accuracy compared with other platforms, we presented a novel approach consisting of multi-locus and ultra-long multiplex amplicon sequencing using the MinION™ MkI DNA sequencer and R9 chemistry that helps to overcome the main disadvantage of this portable sequencing platform. Despite thetechnical issues impairing generation of 2D reads with more per-base accuracy, when processed with adequate alignment filters, the 1D reads obtained this way were sufficient to characterize the microbial species present in each mock community analysed. Improvements in nanopore chemistry, such as minimising base-calling errors and new library protocols able to produce rapid 1D libraries, will

providemore reliable information. Such datawill be useful for more comprehensive and faster specific detection of microbial species and strains in complex ecosystems.

Keywords: MinION; Nanopore sequencer; Ribosomal operon; Ultra-long amplicon sequencing; Microbial diversity; Long-read sequencing

Background

During the last two years, DNA sequencing based on single-molecule technology has completely changed the perception of genomics for scientists working in a wide range of scientific fields. This new perspective is not only supported by the technology itself but also by the affordability of these sequencing instruments. In fact,unprecedentedly, Oxford Nanopore Technologies (ONT) released the first miniaturised and portable DNA sequencer in early 2014, within the framework of the MinION^{TM} Access Programme. As a consequence, certain scientists were initially granted with the MinION™, a USB flash-sized device for DNA sequencing,operated from a computer via USB 3.0. After two years working with the MinION™ device, the scientific community has demonstrated the hugeimpact of this technology on genomic analyses. Recently, the MARC consortium (MinION Analysis and Reference Consortium) has published results related to the study of the reproducibility and global performance of the MinION™ platform. These results indicate that this platform is susceptible of a large stochastic variation, essentially derived from the wet-lab and MinION™ operative methods, but also that variability has minimal impact on data quality [1].

The coordinated and collaborative work and mutual feedback between industry and the scientific community have enabled ONT nanopore technology to develop rapidly towards improving its portable platform for DNA sequencing, minimizing the stochastic variation during DNA library preparation. Moreover the MinION^{TM} operative interface has become more user-friendly. All the above are in addition to the on-going improvement in per-base accuracy of DNA reads derived from the MinION^{TM} device. Consequently, in late Autumn

2015, ONT released MkI, the latest version of MinION^{TM}, and in April 2016the fast mode chemistry (R9) was released, increasing the rate of sensing DNA strands from 30-70 to 280-500 bp/sec and reaching up to 95% of per-base accuracy in 2D reads (Clive G. Brown, CTO ONT, personal communication).

One of the most attractive capabilities of the MinION™ platform is the sequencing and assembly of complete bacterial genomes using exclusivelynanopore reads [2] or through hybrid approaches [3, 4]. Notwithstanding, the MinION™ platform has also been demonstrated useful in other relevant areas including: human genetic variant discovery [5, 6], detection of human pathogens [7, 8], detection of antibiotic resistance [9, 10], and microbial diversity [11, 12]. Regarding the latter, microbial diversity and taxonomic approaches are common and in high demand to analyse the microbiota associated to a wide variety of environment- and human-derived samples. However, these analyses are greatly limited by the short-read strategies commonly employed. Thanks to improvements in the chemistry of the most common, popular sequencing platforms in recent years, it is now possible to characterise microbial communities in detail, down to the family or even genus level, using genetic information derived from roughly 30% (~500nt) of the full 16S rRNA gene. Despite the massive coverage achieved with short-read methods, the limitation in terms of read length means taxonomic assignment at the species level is still unfeasible. Consequently, implementation of long-read sequencing approaches to study larger fragments of marker genes will permit the design of new studies to provide evidence for the central role of precise bacterial species/strains in a great variety of microbial consortia.

With respect to the above, we have recently explored the performance of the MinION™ device. Our study demonstrates that data obtained from sequencing nearly full-length 16S rRNA gene amplicons is feasible to study microbial communities through nanopore technology [11]. We wanted to move a step forward in this type of strategy by designing a multi-locus and ultra-long amplicon sequencing method to study microbial diversity. Accordingly, here we present a study of the 16S, 23S, and the internal transcribed spacer (ITS, that frequently encodes tRNA genes) simultaneous sequencing, using the MinION™ MkI device and R9 chemistry, with prior generation of ~4.5kb DNA fragments by amplifying the nearly full-length operon encoding the two larger ribosomal RNA genes in bacteria, the *rrn* region (*rrn*hereinafter). We have studied the *rrn* of two mock microbial communities, composed of genomic DNA from 20 and 8 different bacterial species, obtained respectively from BEI Resources and ZYMO Research Corp., using the MinION™ sequencing platform.

Data description

Raw data collected in this experiment were obtained as fast5 files using MinKNOW™ v0.51.3.40 (Oxford Nanopore Technologies) after conversion of electric signals into base calls via the Metrichor™ agent v2.40.17 and the Barcoding plus 2D Basecalling RNN for SQK-NSK007 workflow v1.107. Base-called data passing quality control and filtering were downloaded and data was converted to fasta format using the *poRe* package [13]. Fast5 raw data can be accessed at the European Nucleotide Archive (ENA) under the project ID PRJEB15264. Only one data set was generated after a sequencing run of MinION™ MkI.

Analysis

The complete or partial gene sequence of the RNA attached to the small subunit of the ribosome is classically used to perform taxonomy and diversity analysis in complex samples containing hundreds of microbial species. In the case of bacterial species, the 16S rRNA gene is the most widely usedDNA marker for taxonomic identification of a particular species, given the relatively high number of hypervariable regions (V1 to V9) present across its sequence. Nowadays, it is possible to study the complete or almost full-length sequence of the 16S rRNA molecule thanks to single-molecule sequencing approaches [11, 14-16]. The identification of complex microbial communities at species-level with raw data obtained from MinION™ or PacBIO platforms is improving; however, uncertainty in taxonomic assignation is still noteworthy given the high proportion of errors in their reads. While future technical advances may improve the quality of DNA reads generated by third generation sequencing devices, new strategies can also be adopted to enhance the performance of these approaches. Consequently, we postulate that a good example of this is to study a common multi-locus region of the bacterial genome, which enables the simultaneous study of more variable regions and locus arrangements, and the operon encoding the ribosomal RNA. Using a complex sample where hundreds of microbial species are potentially present (DNA from human faeces) we carried out preliminary experiments to amplify the *rrn*. We observed that from the hypothetical configurations envisaged for the *rrn* (Figure 1A), we only obtained a clear amplification using the primerpairs S-D-Bact-0008-c-S-20 and 23S-2241R, indicating that the *rrn* preferentially seems to be transcriptionally arranged as follows: 16S-ITS-23S. A detailed evaluation of the fragment size determined that main PCR products ranged from 4.3 to 5.4kbp (Figure

1B-D), being consistent with the expected size of PCR products amplifying the 16S, ITS, and 23S regions from several microbial species. The next step involved designing a multiplex sequencing approach to try to analyse more than one sample per sequencing run in one flowcell of MinION™; therefore, the primers were re-designed to include a distinctive barcode region at 5´ (Table 1). During PCR of the *rrn* we tagged the amplicon derived from the mock community HM-782D with the barcode *bc01* in a dual manner, whereas the amplicons derived from sample D6305 were tagged with barcode *bc08* in similar way.

For general knowledge, the DNA reads derived from the MinION™ device can be classified into three types: 'template', 'complement', and '2D' reads. The latter,2D reads, are products of aligning and merging sequences from the template (read from leader adapter) and complementreads (a second adapter called hairpin or HP adapter must be generated), produced from the same DNA fragment. These contain a lower error rate, owing to strand comparison and mismatch correction. Following the amplicon library construction and sequencing run, we obtained raw data consisting of 17,038 reads in HDF5 (fast5) format, and almost all were classified as 1D reads. In addition to the technical issues indicative of a bad ligation of the HP adapter, we obtained 93% of reads (~15,900 reads) during the first 16h of run; thus, we obtained lower sequencing performance after re-loading with the second aliquot of the sequencing libraryand extended the run for another 24h (40hin sum). Subsequent to base-calling with Metrichor™ agent, the HDF5 data files were converted into fasta format using the *poRe* [13] package. The fasta sequences were filtered by retaining those between 1500 and 7000nt in length, obtaining at least enough sequence information to compare a DNA sequence equivalent to the 16S rRNA genelength.

After this filtering step, we retained 72% of sequences (12,278) and then we performed the respective barcode splitting. For this purpose, we modified the default parameters of the "split_barcodes.pl" perl script (Oxford Nanopore Technologies) by incorporating the information of the extended barcodes (Table 1), rather than the barcode information alone, and simultaneously increased the stringency parameter to 25 (14 by default). Afterwards the concatenation of reads were obtained from respective forward and reverse extended barcodes, then we retrieved a total of 2,019 (52% from forward and 48% from reverse barcodes) and 1,519 (53% from forward and 47% from reverse barcodes) 1D reads for HM-782D and D6305 mock communities, respectively. Read-mapping was performed against the *rrn* database, compiling more than 22,000 *rrn* regions, retrieved from more than 67,000 genomes available in GenBank (see Availability of supporting data). The taxonomy associated to the best hit based on the alignment score followed by filtering steps (see methods) was used to determine the structure of each mock community. The MinION^{TM} sequencing data produced the microbial structure presented in Figure 2 for the mock communities HM-782D and D6305, respectively.

Figure 2 shows the bacterial species and their respective relative proportions retrieved from the analysis of the mock communities HM-782D and D6305, respectively. With respect to the HM782-D mock community, we were able to recover 20 representative species, accounting for 16 out of 20 species present in that artificial community (Figure 2A). However, the remaining four species that apparently are absent in this community have a close relationship to others detected correctly, namely *Bacillus subtilis, Bacillus thuringensis, Bacillus anthracis,* and *Propionibacterium sp.* Furthermore, we were unable to report the presence of just four species present in HM782-D because proportions of

Rhodobacter sphaeroides and *Actinomyces odontolyticus* were below the predominance threshold (1%), being present in 0.25 and 0.12%, respectively. Previously we have demonstrated that the low levels of 16S reads are a consequence of amplification bias derived from the PCR reaction and not from sequencing itself [11]. In this case, the new primer pair used to generate the ultra-long ampliconswould seem to work more efficiently than those previously used, but apparently they still present issues at bacterial coverage level. When we revised the whole taxonomy contained in our *rrn* database, the compiling of non *rrn* regions for *Deinococcus radiodurans* and *Helicobacter pylori* partially explained the lack of these species in HM-782D analysed by the present approach. However, a new alignment process using individual 16S and 23S rRNA sequences obtained from GenBank and including those for *D. radiodurans* and *H. pylori*, respectively, demonstrated that at least *D. radiodurans* could be identified in a higher proportion than *A. odontolyticus* and *R. sphaeroides*, albeit in a lower proportion than our predominance threshold (data not shown). Regarding the results obtained from the D6305 mock community, we found a total of 10 bacterial species present in this mixed DNA sample, and eight of them matched the expected structure of the community. In this case we were able to recover 100% of the species present in the sample and the two additional members identified also have a close relationship within the *Bacillus* genus, as observed in the HM-782D sample (Figure 2B).

When compared to reference values and proportions theoretically expected for the species present in the two mock communities, we observed some deviations that were greater incertain species. Particularly, in the HM-782D sample the lowest coverage biases were observed for *Actinomyces odontolyticus* (-5.36), *Rhodobacter sphaeroides* (-4.36), and

Enterococcus faecalis (-2.04). This indicates that such species, in addition to *D. radioduran* *s*and *H. pylori*, are more difficult to detect with the primers and PCR used here. By contrast, *Escherichia coli* (1.79) seems to be preferentially amplified, given that this species exhibited the highest positive coverage bias value (Figure 2C). The values obtained for D6305 were more homogeneous, and the lowest coverage bias was observed for *Lactobacillus fermentum* (-2.18) (Figure 2D). Although the low coverage bias for some species can be solved by selecting another pair of primers, the ability to recover almost all of them, at least in a low proportion, in itself represents an important attribute of this approach for inter-sample comparisons.

Another remarkable feature highlighted by our results is the ability to discern reads obtained from both samples despite the high error rate of the 1D reads (ranging between 70 and 87% sequence identity, according to high quality alignments), which make barcoding de-multiplexing difficult. With the configuration and parameters presented here we could efficiently distinguish the reads generated from HM-782D and D6305 amplicons. One example was the ability to discern close species, such as *Lactobacillus gasseri* and *Lactobacillus fermentum*, presented distinctively in HM-782D and D6305 samples, respectively. Also *Salmonella enterica* was determined only in D6305, despite its close relationship with *E. coli* at the 16S and 23S sequence level (close to 100%). Regarding the latter, the multiple sequence alignment built with *rrn* regions from both species was inspected directly distinguishing the ITS as the major source of variation between the two species (data not shown). Indeed, this was corroborated by the comparative analysis performed during the clustering step of the reference samples to create our *rrn* database. The full *rrn* region and its encoded genes were individually clustered at different sequence

identity levels (from 97 to 100%). This analysis indicated that 16S and 23S genes contain a similar level of variation by obtaining a comparable number of clusters (Figure 3). By contrast, the *rrn* region comprising the 16S, ITS, and 23S coding regions exhibits 2-fold more variation than that observed for the 16S molecule alone. Strikingly, the ITS regions showed the highest level of variability when over 3-fold more clusters were produced at 97, 98 and 99% sequence identity. When parsing the genetic information of over 67,000 bacterial genomes, we observed the ITS region frequently encodes one or several tRNA genes. We hypothesized this greater level of variation in the bacterial ITS is directly caused by the variety of tRNA genes encoded in this region, which would even enable different operons from the same bacterial chromosome to be distinguished. Furthermore, the smaller size of this region, together with the high level of sequence variability, would explain the larger number of clusters recovered when compared to the 16S alone.

Discussion

The inventory of microbial species based on 16S rDNA sequencing is frequently used in biomedical research to determine microbial organisms inhabiting the human body and their relationship with disease. Recently, third-generation of DNA sequencing platforms have developed rapidly, facilitating the identification of microbial species and overcoming the read-length issues inherent to second-generation sequencing methods. These advances allow researchers to infer taxonomy and analyse diversity from the almost full-length bacterial 16S rRNA sequence [11, 14-16]. Particularly, the ONT platform deserves special attention given its portability and its fast development since theMinION™ became available in 2014. Notwithstanding, this technology is susceptible to a large stochastic

variation, essentially derived from the wet-lab methods [1]. We corroborated this issue by obtaining a sequencing run where the raw data predominantly consisted of 1D reads as a consequence of the HP adapter ligation failure, despite following the manufacturer's instructions. However, we were able to develop an efficient analysis protocol where the higher read quality offered by R9 chemistry and the updated Metrichor basecaller protocol proved pivotal to obtain 1D reads with a range of identity between 70 and 86%, with sufficient per-base accuracy to successfully perform the taxonomic analyses described herein.

Our preliminary results indicated that the *rrn* region in bacteria preferentially has a unique conformation (with the transcriptional arrangement of 16S-ITS-23S) and we could amplify this ~4.5Kbp region with the selected S-D-Bact-0008-c-S-20 and 23S-2241R primer pair. Once we were able to distinguish the feasibility to amplify the *rrn*, our approach comprised the study of two different mock communities in a multiplex manner, to be combined in one single MinION™ flowcell. By designing the respective forward and reverse primers tagged with specific barcodes recommended by ONT, we were able to retrieve extended barcode-associated reads, in spite of the large proportion of per-base errors contained in these types of reads. Using MinION™ data based on multilocus markers and ultra-long amplicon sequencing, we could reconstruct the structure of two commercially available mock communities. Although the expected proportions of some species in each community exhibited an important coverage bias, we were able to recover 80% (HM-782D) and 100% (D6305) of bacterial species fromthe respective mock communities. Consequently, future analyses should be conducted to find an appropriate PCR approach using primers with a higher coverage for bacterial species.

We have analysed a great amount of genetic information with the aim of compiling a valuable database containing the genetic information for the *rrn* present in over 67,000 draft and complete bacterial genomes. Using this genetic information of the *rrn* and clustered at 100% of sequence identity enabled us to establish a multi-locus marker able to discriminate the taxonomy of two mock communities containing very close species. The latter was possible given that simultaneous analysis of the 16S, ITS, and 23S molecules offered 2-fold more diversity that studying the 16S or 23S sequences separately. Moreover, the ITS was distinguished individually as a highly variable genetic region in terms of sequence and length. Furthermore, it contributes notably to the higher variability observed in the *rrn* region, a fact evidenced in previous studies [17-20]. The accumulation of a larger number of variable sites in the *rrn* region, together with the peculiarity of the ITS to potentially accommodate and encode tRNA genes, are thought to be central to discriminating bacterial species, despite the large proportion of per-base errors contained in MinION™ reads. This idea was supported by the fact that the alignments produced among high quality reads and reference sequences of the *rrn* database had a length average of 2,655 and 2,918 bases for HM-782D and D6305, respectively. This latter is indicative that ITS genetic information was predominantly considered in our analysis no matter if reads were produced from the 16S or 23S edges of *rrn* amplicons. We expect this type of analysis will likely become more accurate over time as nanopore chemistry improves in future technology, with the concomitant increase in throughput, which is pivotal to disclose the hundreds of species present in complex microbial communitiesfor analysis in human or environmental studies. Therefore, the multi-locus, ultra-long and multiplex methods described here represent a promising analysis routine for microbial and pathogen identification, relying on the sequence variation accumulated in approximately 5kbp of

DNA, roughly accounting for the assessment of 1.25% of an average bacterial genome (~4Mbp).

Methods

Bacterial DNA and rrnamplicons

The complex DNA sample for preliminary studies of *rrn* region arrangement consisted of DNA isolated from faeces, kindly donated by a healthy volunteer upon informed consent. An aliquot of 200 mg of human faeces was used to isolate microbial DNA using the QIAamp DNA Stool Mini Kit (Qiagen) and following the manufacturer's instructions. Finally, DNA was eluted in 100 μL nuclease-free water and a DNA aliquot at 20 ng/μL was prepared for PCR reaction using the primer pairs S-D-Bact-0008-c-S-20 and 23S-2241R or 23S-129F and S-D-Bact-1391-a-A-17 for testing configurations shown in Figure 1A (Table 1). The band size was analysed using the Java-based GelAnalyzer tool (www.gelanalyzer.com). Genomic DNA for the reference mock microbial communities was kindly donated by BEI Resources (http://www.beiresources.org) and ZYMO Research Corp (http://www.zymoresearch.com). The composition of the mock communities was as follows: i) HM-782D is a genomic DNA mixture of 20 bacterial species containing equimolar ribosomal RNA operon counts (100,000 copies per organism per μL), as indicated by the manufacturer; and ii) ZymoBIOMICS Cat No. D6305 (D6305 hereinafter) is a genomic DNA mixture of eight bacterial species (and two fungal species) presented in equimolar amounts ofDNA. According to manufacturers' instructions, 1 μL of DNA from each mock community was used to amplify all the genes contained in the *rrn*. DNA was amplified in triplicate by 27 PCR cycles at 95ºC for 30 s, 49ºC for 15 s, and 72ºC for 210 s.

Phusion High-Fidelity Taq Polymerase (Thermo Scientific) and the primers S-D-Bact-0008-c-S-20 (mapping on 5' of 16S gene) and 23S-2241R (mapping on 3' of 23S gene), which target a wide range of bacterial 16S rRNA genes [21, 22]. As we wished to multiplex the sequencing of both mock communities into one single MinION™ flowcell, we designed a dual-barcode approach where respective primers were synthesized and fusedwith two different barcodes recommended by ONT (Table 1). Amplicons consisted of ~4.5kbp blunt-end fragments, which were purified using the Illustra GFX PCR DNA and Gel Band Purification Kit (GE Healthcare). Amplicon DNA was quantified using a Qubit 3.0 fluorometer (Life Technologies).

Amplicon DNA library preparation

The Genomic DNA Sequencing Kit SQK-MAP006 was ordered from ONT and used to prepare the amplicon library for loading into the MinION™. Approximately 0.9μg of amplicon DNA (0.3 per mock community plus 0.3 μg of an extra query sample) were processed for end repair using the NEBNextUltra II End Repair/dA-tailing Module (New England Biolabs), and followed by purification using Agencourt AMPure XP beads (Beckman Coulter) and washing twice with 1 volume of fresh 70% ethanol. Subsequently, and according to the manufacturer’s suggestions, we used 0.2 pmol of the purified amplicon DNA (~594 ng, assuming fragments of 4.5kbp in length) to perform the adapter ligation step. Ten μL of adapter mix, 2 μL of HP adapter, and 50 μl of Blunt/TA ligase master mix (New England Biolabs) were added in that order to the 38μlend-repairedamplicon DNA. The reaction was incubated at room temperature for 15 minutes, 1 μL HP Tether was added and incubated for an additional 10 minutes at room temperature.

The adapter-ligated amplicon was recovered using MyOne C1-beads (Life Technologies) and rinsed with washing buffer provided with the Genomic DNA Sequencing Kit SQK-MAP006 (Oxford Nanopore Technologies). Finally, the sample was eluted from the MyOne C1-beads by adding 25 μL of elution buffer and incubating for 10 minutes at 37°C before pelleting in a magnetic rack.

Flowcell set-up

A brand new, sealed R9flowcell was acquired from ONT and stored at 4°C before use. The flowcell was fitted to the MinION™ MkI prior to loading the sequencing mix, ensuring good thermal contact. The R9flowcell was primed twice using 71 μL premixed nuclease-free water, 75 μL 2x running buffer, and 4μLfuel mix. At least 10 minutes were required to equilibrate the flowcell before each round of priming and before final DNA library loading.

Amplicon DNA sequencing

The sequencing mix was prepared with 59μl nuclease-free water, 75 μl2x running buffer, 12μL DNA library, and 4 μL fuel mix. A standard 48-hour sequencing protocol was initiated using the MinKNOW™ v0.51.3.40. Base-calling was performed through data transference using the Metrichor™ agent v2.40.17 and the Barcoding plus 2D Basecalling RNN for SQK-NSK007 workflow v1.107. During the sequencing run, one additional freshly diluted aliquot of DNA library was loaded after 16 hours of initial input. The rawsequencing data derived from the two mock communities studied here was expected to account two-thirds of the data produced by the R9 flowcell used.

The rrn database

We built a database containing the genetic information for the 16S and 23S rRNA genes and the ITS sequence in all the complete and draft bacterial genomes available in the NCBI database (ftp://ftp.ncbi.nlm.nih.gov/genomes/genbank/bacteria). A total of 67,199 genomes were analysed by downloading the "*fna*" files and parsing for rRNAgenes into the respective "*gff*" annotation file. Chromosome coordinates for *rrn* regions were parsed and used to extract such a DNA sequences from complete chromosomes or DNA contigs assembled. The resulting *rrn* sequences were analysed and the length distribution was assessed (Figure 1). We retrieved a total of 47, 698 *rrn* sequences with an average of 4,993nt in length. By selecting the size distribution equal to the 99th percentile (two-sided), we discarded potential incomplete or aberrant annotated *rrn* sequences and observed that *rrn* sequences can be found between 4,196 and 5,790nt; under these boundaries, our *rrn* database finally accounted for a total of 46,920 sequences.Equivalent databases were built by parsing the respective *rrn* sequences with the RNammer tool to discriminate the 16S, ITS, and 23S rRNA sequences [23]. To remove the level of redundancy of our *rrn* database and to maintain the potential discriminatory power at strain level, we performed clustering analysis using USEARCH v8 tool for sequence analysis and the option *-otu_radius_pct* equal 0 [24], thus obtaining a total of 22,350 reference sequences. For comparative aims, the *rrn* database and the 16S, ITS, and 23S databases were also analysed using the option -*otu_radius_pct* with values ranging from 1 to 3. For accessing to *rrn* database and the respective species annotation, see Availability of supporting data.

Data analysis

Read-mapping was performed using the LAST aligner v.189 [25] with parameters -q1 -b1 -Q0 -a1 -r1. Each 1D read was compared in a competitive way against the entire *rrn* database and the best hit was selected by obtaining the highest alignment score. Alignment length as well as alignment coordinates in target and query sequences were parsed from the LAST output and the sequence identity between matched regions was calculated using the python *Levenshtein* distance package. High quality alignments were selected by filtering out those with identity values up to the 50th percentile of the distribution of identity values of all reads per sample (~69%). Therefore, taxonomy assignment was based exclusively on alignments with ≥ 70% identity.Basic stats, distributions, filtering, and comparisons were performed in R v3.2.0 (https://cran.r-project.org). For relative quantification of species the singletons were removed and the microbial species considered to be predominantly present in the mock communities were those with a relative a proportion ≥ 1%. The coverage bias was calculated by obtaining fold-change (Log_2) of species-specific read counting against the expected (theoretical) average for the entire community according to information provided by the manufacturers.

Availability of supporting data

Accessions for the *rrn* database containing the reference sequences for alignments and taxonomic annotation is available at https://github.com/alfbenpa/rrn_db. The code source of the original *split_barcodes.pl* perl script is available at https://github.com/nanoporetech/barcoding/releases/tag/1.0.0 with ONT copyright.

Abbreviations

EC , European Commission; ENA, European Nucleotide Archive; HDF, Hierarchical Data Format; ITS, internal transcribed spacer; NCBI, National Center for Biotechnology Information; ONT, Oxford Nanopore Technologies; PCR, Polymerase Chain Reaction; rDNA, DNA encoding for the Ribosomal RNA; rRNA, Ribosomal RNA;*rrn*, the DNA region containing the 16S and 23S bacterial rRNA genes and its respective ITS region; USB, Universal Serial Bus.

Competing interests

ABP is part of the MinION™ Access Programme (MAP).

Authors' contributions

ABP and YS designed the study and managed the project. ABP performed the experiments, analysed and managed the data. ABP draft the manuscript. Both authors read and approved the final manuscript.

Acknowledgements

This work and the contract to ABP is supported by the European Union's Seventh Framework Program under the grant agreement nº 613979 (MyNewGut).

References

1. Ip CL, Loose M, Tyson JR, de Cesare M, Brown BL, Jain M, Leggett RM, Eccles DA, Zalunin V, Urban JM *et al*: **MinION Analysis and Reference Consortium: Phase 1 data release and analysis**. *F1000Res* 2015, **4**:1075.
2. Loman NJ, Quick J, Simpson JT: **A complete bacterial genome assembled de novo using only nanopore sequencing data**. *Nat Methods* 2015, **12**(8):733-735.
3. Karlsson E, Larkeryd A, Sjodin A, Forsman M, Stenberg P: **Scaffolding of a bacterial genome using MinIONnanopore sequencing**. *Sci Rep* 2015, **5**:11996.
4. Risse J, Thomson M, Patrick S, Blakely G, Koutsovoulos G, Blaxter M, Watson M: **A single chromosome assembly of Bacteroidesfragilis strain BE1 from Illumina and MinIONnanopore sequencing data**. *Gigascience*2015, **4**:60.
5. Ammar R, Paton TA, Torti D, Shlien A, Bader GD: **Long read nanopore sequencing for detection of HLA and CYP2D6 variants and haplotypes**. *F1000Res* 2015, **4**:17.
6. Norris AL, Workman RE, Fan Y, Eshleman JR, Timp W: **Nanopore sequencing detects structural variants in cancer**. *Cancer BiolTher*2016:1-8.
7. Greninger AL, Naccache SN, Federman S, Yu G, Mbala P, Bres V, Stryke D, Bouquet J, Somasekar S, Linnen JM *et al*: **Rapid metagenomic identification of viral pathogens in clinical samples by real-time nanopore sequencing analysis**. *Genome Med* 2015, **7**(1):99.
8. Kilianski A, Haas JL, Corriveau EJ, Liem AT, Willis KL, Kadavy DR, Rosenzweig CN, Minot SS: **Bacterial and viral identification and differentiation by amplicon sequencing on the MinIONnanopore sequencer**. *Gigascience*2015, **4**:12.
9. Ashton PM, Nair S, Dallman T, Rubino S, Rabsch W, Mwaigwisya S, Wain J, O'Grady J: **MinIONnanopore sequencing identifies the position and structure of a bacterial antibiotic resistance island**. *Nat Biotechnol*2015, **33**(3):296-300.
10. Judge K, Harris SR, Reuter S, Parkhill J, Peacock SJ: **Early insights into the potential of the Oxford NanoporeMinION for the detection of antimicrobial resistance genes**. *J AntimicrobChemother*2015, **70**(10):2775-2778.
11. Benitez-Paez A, Portune KJ, Sanz Y: **Species-level resolution of 16S rRNA gene amplicons sequenced through the MinION portable nanopore sequencer**. *Gigascience*2016, **5**:4.
12. Li C, Cheng KR, Boey JHE, Ng HQA, Wilm A, Nagarajan N: **INC-Seq: Accurate single molecule reads using nanopore sequencing**. *bioRxiv*2016:doi: http://dx.doi.org/10.1101/038042
13. Watson M, Thomson M, Risse J, Talbot R, Santoyo-Lopez J, Gharbi K, Blaxter M: **poRe: an R package for the visualization and analysis of nanopore sequencing data**. *Bioinformatics* 2014, **31**(1):114-115.
14. Li C, Chng KR, Boey EJ, Ng AH, Wilm A, Nagarajan N: **INC-Seq: accurate single molecule reads using nanopore sequencing**. *Gigascience*2016, **5**(1):34.
15. Schloss PD, Jenior ML, Koumpouras CC, Westcott SL, Highlander SK: **Sequencing 16S rRNA gene fragments using the PacBio SMRT DNA sequencing system**. *PeerJ*2016, **4**:e1869.

16. Shin J, Lee S, Go MJ, Lee SY, Kim SC, Lee CH, Cho BK: **Analysis of the mouse gut microbiome using full-length 16S rRNAamplicon sequencing**. *Sci Rep* 2016, **6**:29681.

17. Fernandez J, Avendano-Herrera R: **Analysis of 16S-23S rRNA gene internal transcribed spacer of Vibrio anguillarum and Vibrio ordalii strains isolated from fish**. *FEMS MicrobiolLett*2009, **299**(2):184-192.

18. Maslunka C, Gurtler V, Seviour R: **Unusual features of the sequences of copies of the 16S-23S rRNA internal transcribed spacer regions of Acinetobacterbereziniae, Acinetobacterguillouiae and Acinetobacterbaylyi arise from horizontal gene transfer events**. *Microbiology* 2015, **161**(Pt 2):322-329.

19. Stewart FJ, Cavanaugh CM: **Intragenomic variation and evolution of the internal transcribed spacer of the rRNA operon in bacteria**. *J MolEvol*2007, **65**(1):44-67.

20. Tambong JT, Xu R, Bromfield ES: **Intercistronic heterogeneity of the 16S-23S rRNA spacer region among Pseudomonas strains isolated from subterranean seeds of hog peanut (Amphicarpabracteata)**. *Microbiology* 2009, **155**(Pt 8):2630-2640.

21. Hunt DE, Klepac-Ceraj V, Acinas SG, Gautier C, Bertilsson S, Polz MF: **Evaluation of 23S rRNA PCR primers for use in phylogenetic studies of bacterial diversity**. *Appl Environ Microbiol*2006, **72**(3):2221-2225.

22. Klindworth A, Pruesse E, Schweer T, Peplies J, Quast C, Horn M, Glockner FO: **Evaluation of general 16S ribosomal RNA gene PCR primers for classical and next-generation sequencing-based diversity studies**. *Nucleic Acids Res* 2012, **41**(1):e1.

23. Lagesen K, Hallin P, Rodland EA, Staerfeldt HH, Rognes T, Ussery DW: **RNAmmer: consistent and rapid annotation of ribosomal RNA genes**. *Nucleic Acids Res* 2007, **35**(9):3100-3108.

24. Edgar RC: **Search and clustering orders of magnitude faster than BLAST**. *Bioinformatics* 2010, **26**(19):2460-2461.

25. Frith MC, Hamada M, Horton P: **Parameters for accurate genome alignment**. *BMC Bioinformatics* 2010, **11**:80.

Figure legends

Figure 1. Organization of the *rrn* region in bacteria. A - hypothetical transcriptional arrangements expected for *rrn* and tested experimentally using two sets of primer pairs (see small arrows drawn in each configuration). B - Agarose gel electrophoresis of PCR reactions performed under the two hypothetical arrangements of *rrn*; lanes: 1) 1kb ruler (Fermentas), 2) PCR reaction from the top configuration in panel A, 3) PCR reaction from the bottom configuration in panel A. The GelAnalyser Java application was used to perform theband size analysis of the 1kb ruler standard (C) and the amplicons obtained from human faecal DNA (D).

Figure 2. Microbial structure of the mock communities. A and B - microbial species and respective relative proportions determined to be present in the HM-782D and D6305 mock communities, respectively, following the analysis of raw data obtained from *rrn* amplicon sequencing in the MinION^{TM}. C and D - Comparative analysis of the expected microbial species and proportions against the data obtained after mapping of reads generated by a 4.5kbp amplicon PCR and sequenced in MinION^{TM} device, for HM-782D and D6305 respectively.

Figure 3. Variability of the *rrn* region and its functional domains. The *rrn* database compiled after parsing more than 67,000 draft and complete bacterial genomes was assessed by clustering analysis at different levels of sequence identity: 97 (white bars), 98 (light grey bars), 99 (dark grey bars), and 100% (black bars). For comparative aims, the functional DNA sequences encoded into the *rrn* region were also individually studied. The fold numbers located at the top of respective ITS, 23S and *rrn* bars indicate the proportion of clusters found and normalized to those found in the 16S.

Table Click here to download Table Table 1.docx

Table 1. Barcodes and primers used to generate amplicon libraries.

Sample	Barcode	Primer	Barcode extended[1]
**HM-782D**	(bc01)<br>GGTGCTGAAGAAAGTTGTCGGTGTCTTTGTGTTAACCT	(S-D-Bact-0008-c-S-20)<br>AGAGTTTGATCMTGGCTCAG	(bc01F)<br>GGTGCTGAAGAAAGTTGTCGGTGTCTTTGTGTTAACCTAGAGTTTGATCMTGGCTCAG
	(bc01)<br>GGTGCTGAAGAAAGTTGTCGGTGTCTTTGTGTTAACCT	(23S-2241R)<br>ACCGCCCCAGTHAAACT	(bc01R)<br>GGTGCTGAAGAAAGTTGTCGGTGTCTTTGTGTTAACCTACCGCCCCAGTHAAACT
**D6503**	(bc08)<br>GGTGCTGTTCAGGGAACAAACCAAGTTACGTTTAACCT	(S-D-Bact-0008-c-S-20)<br>AGAGTTTGATCMTGGCTCAG	(bc08F)<br>GGTGCTGTTCAGGGAACAAACCAAGTTACGTTTAACCTAGAGTTTGATCMTGGCTCAG
	(bc08)<br>GGTGCTGTTCAGGGAACAAACCAAGTTACGTTTAACCT	(23S-2241R)<br>ACCGCCCCAGTHAAACT	(bc08R)<br>GGTGCTGTTCAGGGAACAAACCAAGTTACGTTTAACCTACCGCCCCAGTHAAACT
**Other primers used**			
**Human fecal DNA**		(S-D-Bact-1391-a-A-17)<br>GACGGGCGGTGWGTRCA	
		(23S-129F)<br>CYGAATGGGRVAACC	

1 Underlined sequences correspond with the barcode sequence

Figure1

Click here to download Figure Figure1.pdf

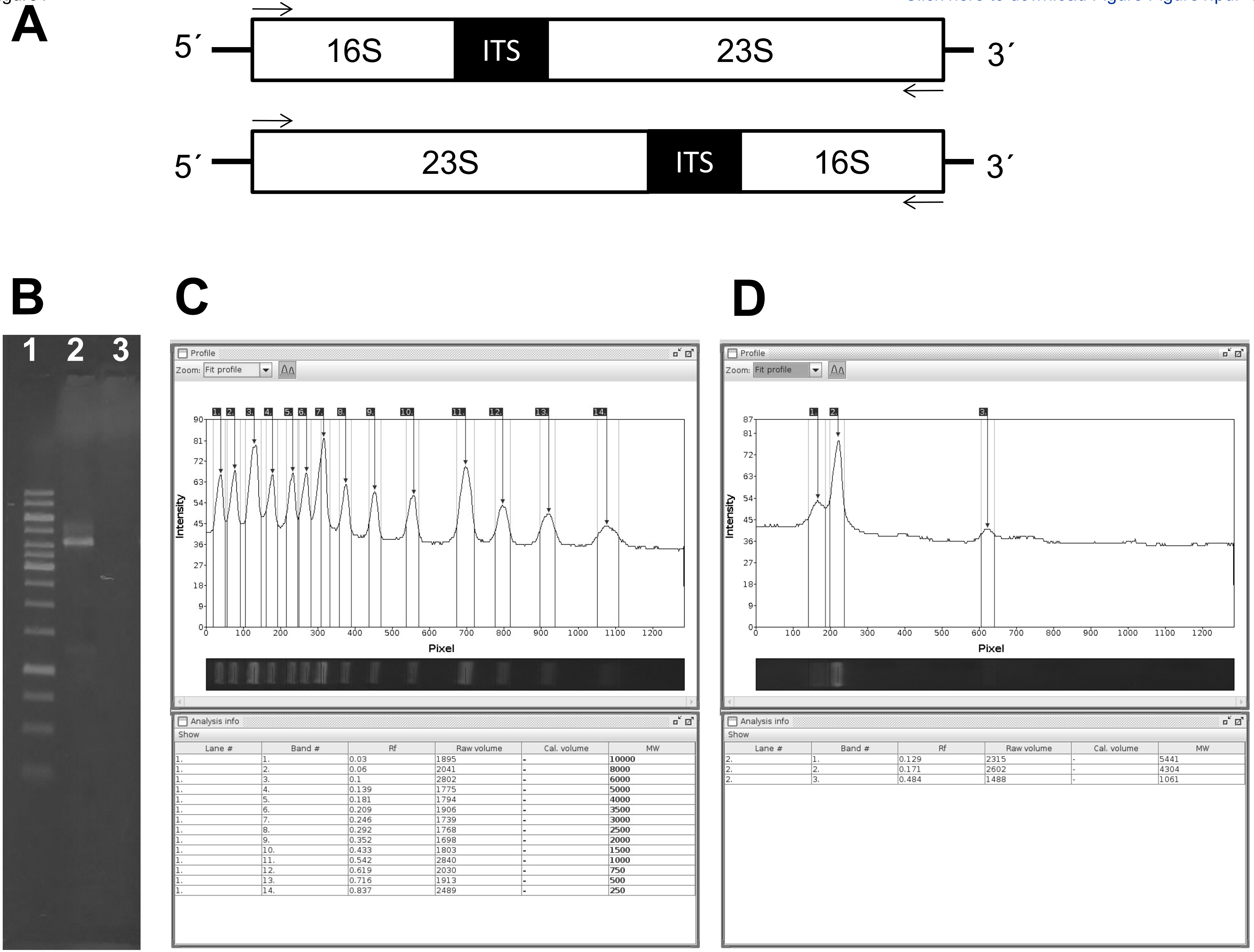

Lane #	Band #	Rf	Raw volume	Cal. volume	MW
1.	1.	0.03	1895	-	10000
1.	2.	0.06	2041	-	8000
1.	3.	0.1	2802	-	6000
1.	4.	0.139	1775	-	5000
1.	5.	0.181	1794	-	4000
1.	6.	0.209	1906	-	3500
1.	7.	0.246	1739	-	3000
1.	8.	0.292	1768	-	2500
1.	9.	0.352	1698	-	2000
1.	10.	0.433	1803	-	1500
1.	11.	0.542	2840	-	1000
1.	12.	0.619	2030	-	750
1.	13.	0.716	1913	-	500
1.	14.	0.837	2489	-	250

Lane #	Band #	Rf	Raw volume	Cal. volume	MW
2.	1.	0.129	2315	-	5441
2.	2.	0.171	2602	-	4304
2.	3.	0.484	1488	-	1061

Figure2 Click here to download Figure Figure2.pdf

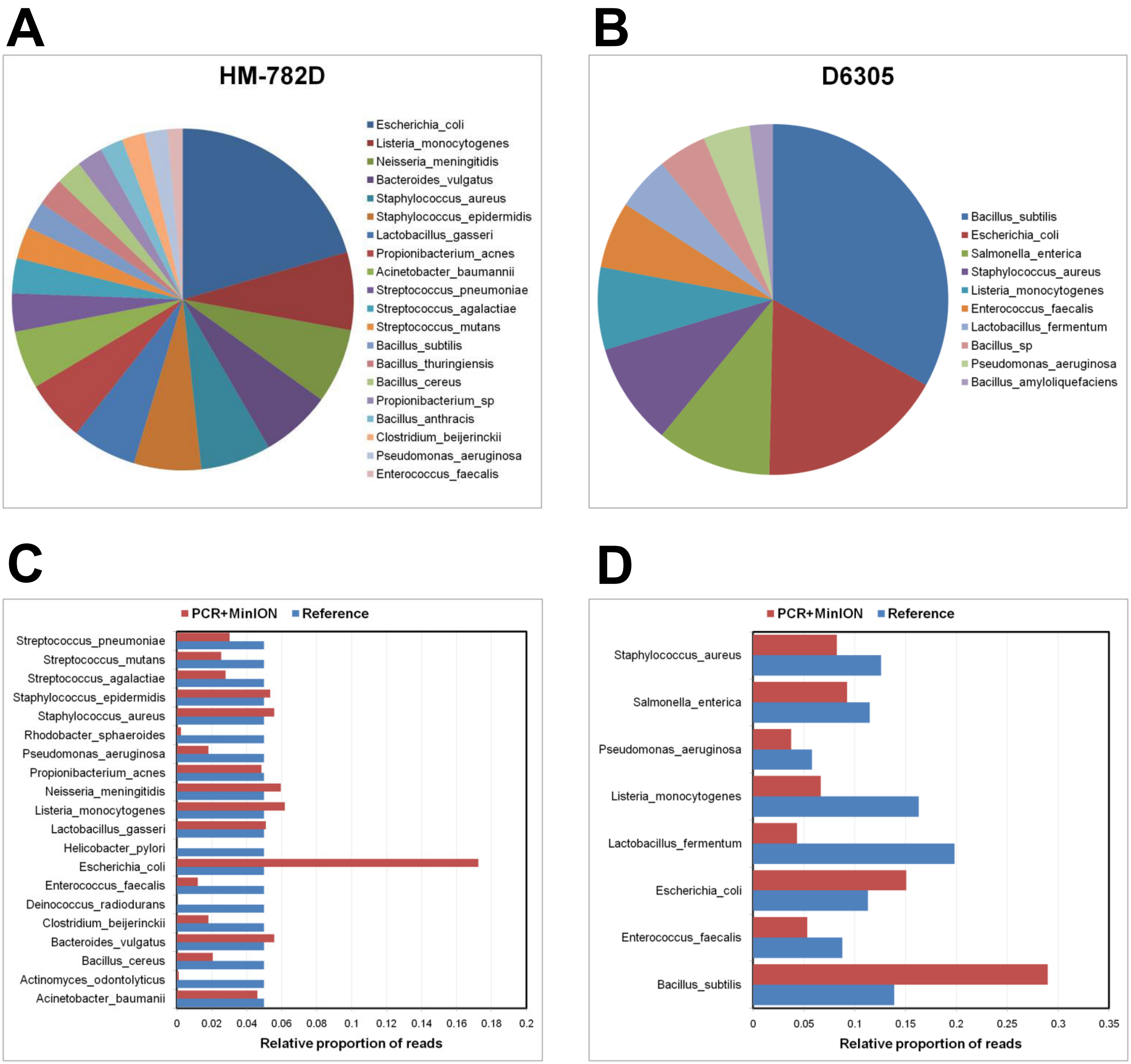

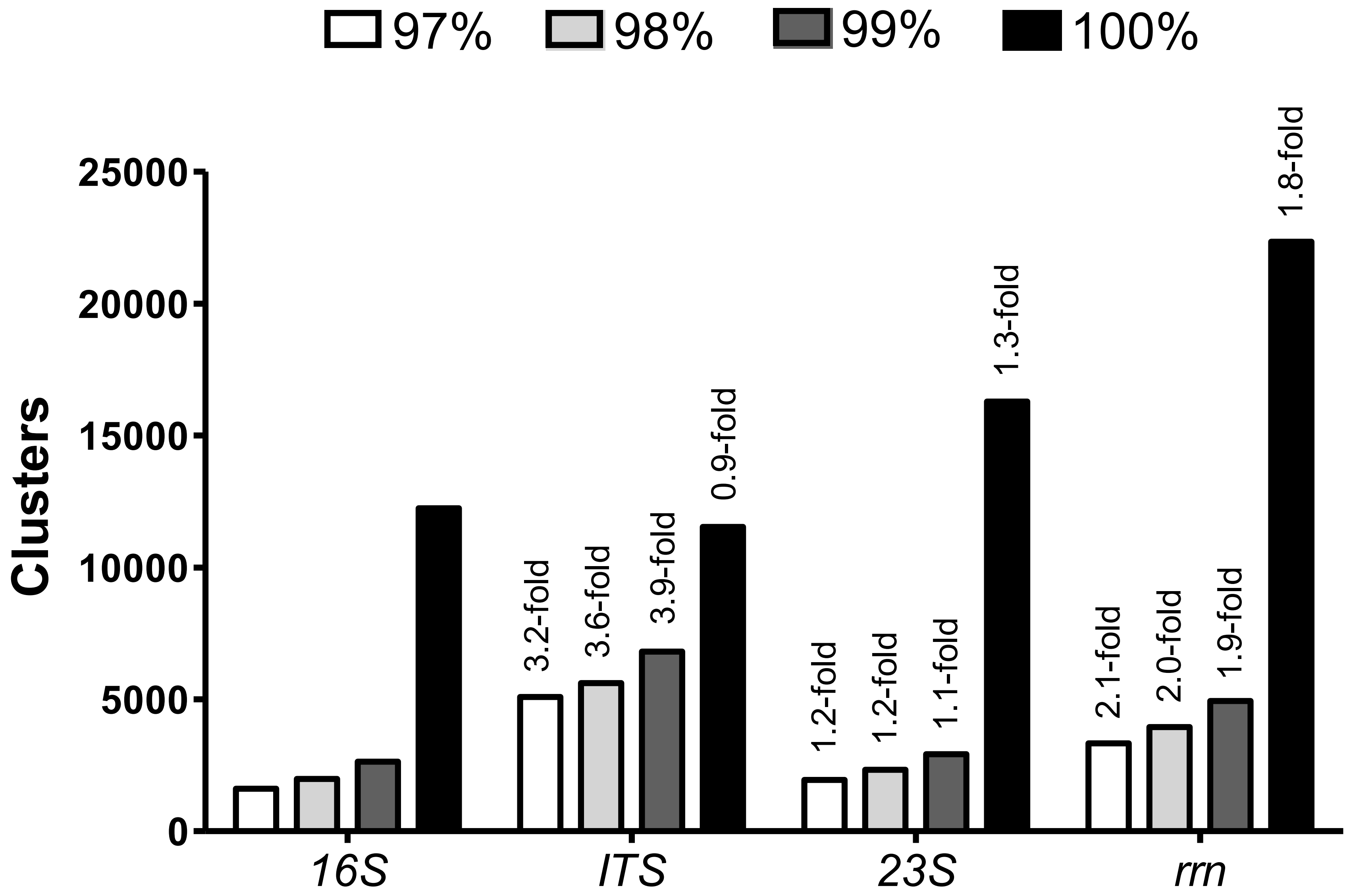
97%
98%
99%
100%
Clusters
25000
20000
15000
10000
5000
0
16S
ITS
23S
rrn
3.2-fold
3.6-fold
3.9-fold
0.9-fold
1.2-fold
1.2-fold
1.1-fold
1.3-fold
2.1-fold
2.0-fold
1.9-fold
1.8-fold

Personal Cover Click here to download Personal Cover Cover_letter.pdf

MINISTERIO
DE ECONOMÍA
Y COMPETITIVIDAD

CSIC
CONSEJO SUPERIOR DE INVESTIGACIONES CIENTÍFICAS

INSTITUTO DE AGROQUÍMICA Y
TECNOLOGÍA DE ALIMENTOS (IATA)

Laurie Goodman Ph.D.
Editor in Chief
GigaScience Journal

Subject: Manuscript submission

Dear Dr Goodman,

Please find attached our manuscript entitled "**Multi-locus and ultra-long amplicon sequencing approach to study microbial diversity at species level using the MinION^{TM} portable nanopore sequencer**" which we would like you to consider for publication in the *GigaScience Journal*. With the submission of this manuscript I would like to undertake that the above mentioned manuscript has not been published elsewhere, accepted for publication elsewhere or under editorial review for publication elsewhere; and that my Institute's (IATA-CSIC) representative is fully aware of this submission.

For the Editor(s) who will handle this document, I would like to disclose the following information about the manuscript:
In the post-genomic era, analysis of the great amount of genetic information derived from microbial communities have permitted to explore their diversity and genetic variability, characteristics that help to disclose certain bacterial groups associated with healthy status and pathogenicity then gaining insights in the microbe-host co-evolution. Nowadays, large scale analyses are needed with the aim of unraveling the complete repertoire of microbes present in different human- and environmental-associated niches. To date, the paired-end short reads approaches for massive sequencing permits the analysis of sequence information of roughly 30% (~500nt) of the 16S rDNA, leaving taxonomic assignment of reads at the species level elusive. Therefore, implementation of long-read sequencing approaches to study the 16S rRNA genes should be determinant to design new studies conducted to evidence the real and central role of precise bacterial species in a great variety of microbial consortia.

Although some efforts have been done in order to assess the almost-full bacteria 16S DNA sequence in order to improve the taxonomy identification in microbial communities, we have moved a step forward to set a new approach that include a multi-locus design to study a consensus bacterial chromosome region highly variable in terms of sequence and gene content. Consequently, in this study, we have explored again the scope of MinION^{TM} into microbial diversity studies by using an ultra-long amplicon sequencing methodology to study simultaneously the 16S, ITS, and 23S regions of the DNA obtained from two different mock bacterial communities in multiplex manner. The usage of MinION^{TM} platform allowed the acquisition of 1D reads of moderate quality which were enough to reconstruct efficiently the microbial community structure artificially compiled into the HM-782D and D6305 commercial DNAs.

To our knowledge, this is the first report to perform a microbial diversity study comparing such level of genetic information derived from a single PCR reaction and sequenced in multiplex manner in a third generation sequencing device. As a consequence, we anticipated a large impact to the scientific community working on this field as the information we provide and retrieved from the nanopore portable DNA sequencer will serve to better describe the diversity of human- or environmental-derived microbial communities.

Thanks for taking this into consideration.

Best wishes,

Alfonso Benítez Páez Ph.D.
Corresponding Author
Agrochemistry and Food Technology Institute (IATA-CSIC)
abenitez@iata.csic.es

info@iata.csic.es

Avda. Agustí Escardino, 7
46980 PATERNA
(VALENCIA) ESPAÑA
TEL.: 963 90 00 22
FAX: 963 63 63 01
