# Supplementary figures and images for: Multi-locus and long amplicon sequencing approach to study microbial diversity at species level using the MinION™ portable nanopore sequencer
Source: Gigascience. 2017 Jun 12;6(7):1–12. doi: 10.1093/gigascience/gix043 (PMC5534310; doi:10.1093/gigascience/gix043)

## HM782D

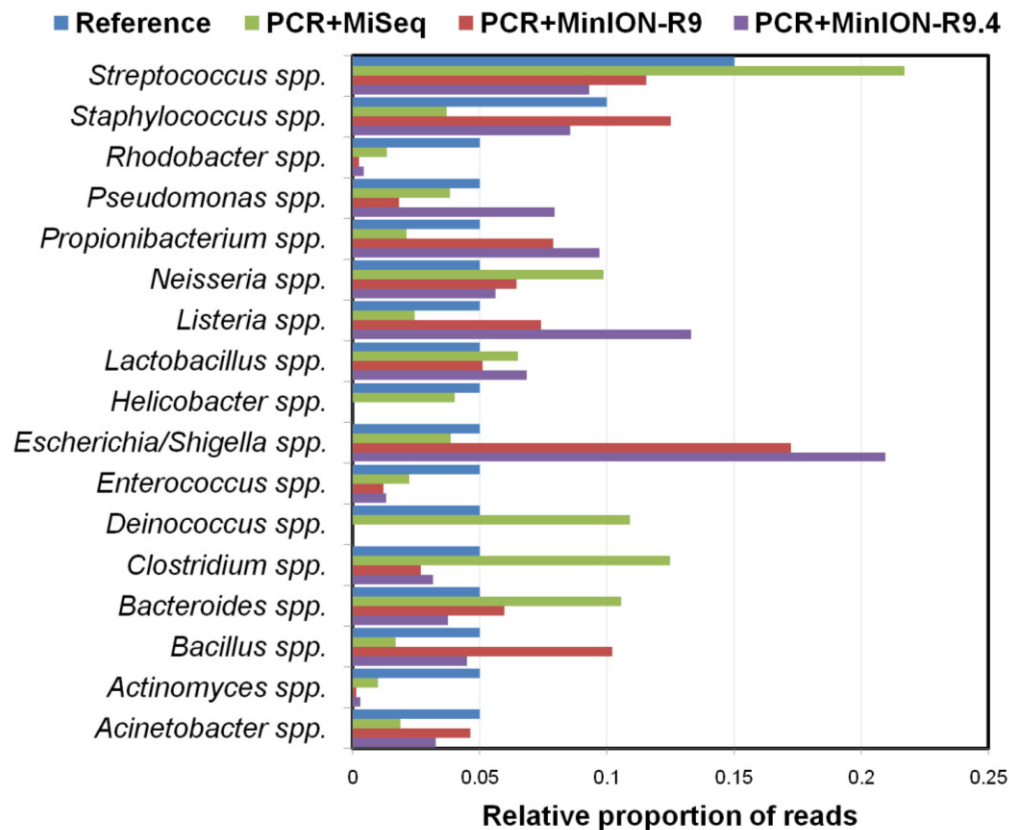

## D6305

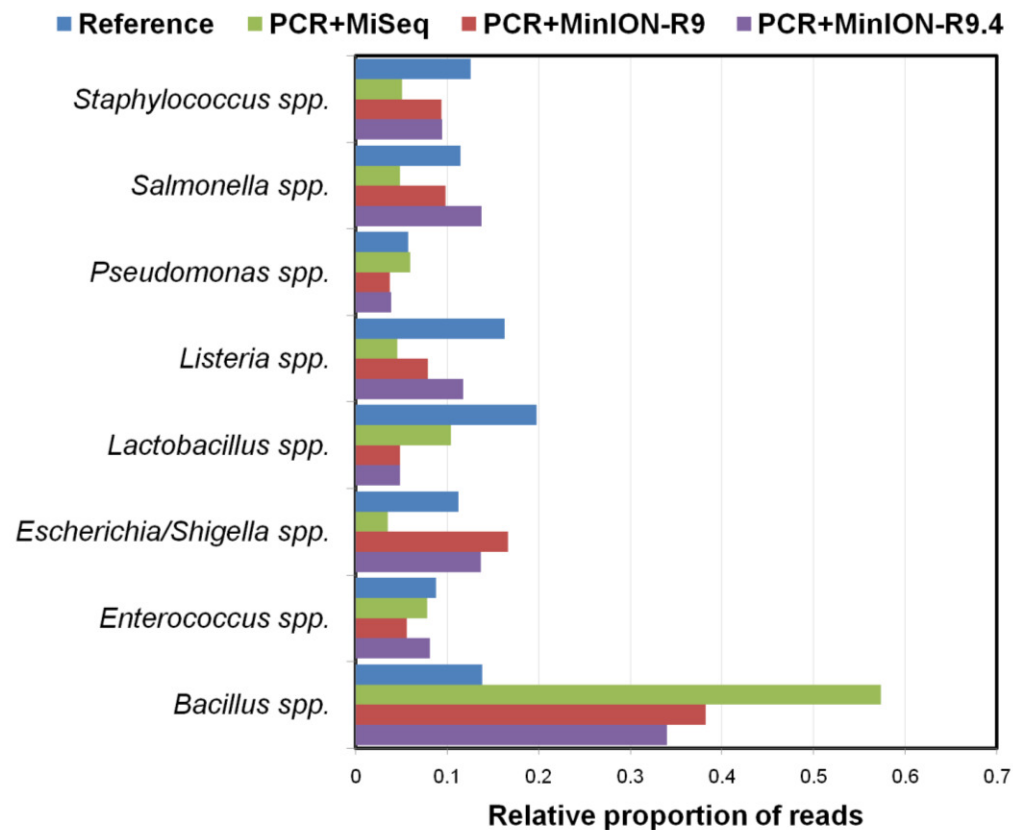

Supplement: Supplementary_Material_1.pdf [file gix043_Supplementary_Material_1.pdf]
